# Assessing Statewide All-Cause Future One-Year Mortality: Prospective Study With Implications for Quality of Life, Resource Utilization, and Medical Futility

**DOI:** 10.2196/10311

**Published:** 2018-06-04

**Authors:** Yanting Guo, Gang Zheng, Tianyun Fu, Shiying Hao, Chengyin Ye, Le Zheng, Modi Liu, Minjie Xia, Bo Jin, Chunqing Zhu, Oliver Wang, Qian Wu, Devore S Culver, Shaun T Alfreds, Frank Stearns, Laura Kanov, Ajay Bhatia, Karl G Sylvester, Eric Widen, Doff B McElhinney, Xuefeng Bruce Ling

**Affiliations:** ^1^ School of Management Zhejiang University Hangzhou China; ^2^ Department of Surgery Stanford University Stanford, CA United States; ^3^ HBI Solutions Inc Palo Alto, CA United States; ^4^ Department of Cardiothoracic Surgery Stanford University Stanford, CA United States; ^5^ Clinical and Translational Research Program Betty Irene Moore Children's Heart Center Lucile Packard Children’s Hospital Palo Alto, CA United States; ^6^ Department of Health Management Hangzhou Normal University Hangzhou China; ^7^ China Electric Power Research Institute Beijing China; ^8^ HealthInfoNet Portland, ME United States; ^9^ Department of Pediatrics Stanford University Stanford, CA United States; ^10^ Department of Epidemiology and Health Statistics School of Public Health, School of Medicine Zhejiang University Hangzhou China

**Keywords:** One-year mortality risk prediction, electronic medical records, quality of life, healthcare resource utilization, social determinants

## Abstract

**Background:**

For many elderly patients, a disproportionate amount of health care resources and expenditures is spent during the last year of life, despite the discomfort and reduced quality of life associated with many aggressive medical approaches. However, few prognostic tools have focused on predicting all-cause 1-year mortality among elderly patients at a statewide level, an issue that has implications for improving quality of life while distributing scarce resources fairly.

**Objective:**

Using data from a statewide elderly population (aged ≥65 years), we sought to prospectively validate an algorithm to identify patients at risk for dying in the next year for the purpose of minimizing decision uncertainty, improving quality of life, and reducing futile treatment.

**Methods:**

Analysis was performed using electronic medical records from the Health Information Exchange in the state of Maine, which covered records of nearly 95% of the statewide population. The model was developed from 125,896 patients aged at least 65 years who were discharged from any care facility in the Health Information Exchange network from September 5, 2013, to September 4, 2015. Validation was conducted using 153,199 patients with same inclusion and exclusion criteria from September 5, 2014, to September 4, 2016. Patients were stratified into risk groups. The association between all-cause 1-year mortality and risk factors was screened by chi-squared test and manually reviewed by 2 clinicians. We calculated risk scores for individual patients using a gradient tree-based boost algorithm, which measured the probability of mortality within the next year based on the preceding 1-year clinical profile.

**Results:**

The development sample included 125,896 patients (72,572 women, 57.64%; mean 74.2 [SD 7.7] years). The final validation cohort included 153,199 patients (88,177 women, 57.56%; mean 74.3 [SD 7.8] years). The c-statistic for discrimination was 0.96 (95% CI 0.93-0.98) in the development group and 0.91 (95% CI 0.90-0.94) in the validation cohort. The mortality was 0.99% in the low-risk group, 16.75% in the intermediate-risk group, and 72.12% in the high-risk group. A total of 99 independent risk factors (n=99) for mortality were identified (reported as odds ratios; 95% CI). Age was on the top of list (1.41; 1.06-1.48); congestive heart failure (20.90; 15.41-28.08) and different tumor sites were also recognized as driving risk factors, such as cancer of the ovaries (14.42; 2.24-53.04), colon (14.07; 10.08-19.08), and stomach (13.64; 3.26-86.57). Disparities were also found in patients’ social determinants like respiratory hazard index (1.24; 0.92-1.40) and unemployment rate (1.18; 0.98-1.24). Among high-risk patients who expired in our dataset, cerebrovascular accident, amputation, and type 1 diabetes were the top 3 diseases in terms of average cost in the last year of life.

**Conclusions:**

Our study prospectively validated an accurate 1-year risk prediction model and stratification for the elderly population (≥65 years) at risk of mortality with statewide electronic medical record datasets. It should be a valuable adjunct for helping patients to make better quality-of-life choices and alerting care givers to target high-risk elderly for appropriate care and discussions, thus cutting back on futile treatment.

## Introduction

Many patients with advanced cancer would prefer to be cared for and die at home. However, among the 50% to 70% of patients with a terminal illness who prefer to be cared for and die at home, only about 25% have a home death, and more than 50% die in the hospital [1]. Nearly a third of Americans who die after age 65 years will have spent time in an intensive care unit in their final 3 months of life, and almost a fifth undergo surgery in their last month [2]. Even more, a disproportionate amount of health care resources and expenditures are spent on patients who are terminally ill [3]. Health care experts estimate that one-quarter of all Medicare costs—US $150 billion annually—goes to treating patients in their last year of life [4].

Despite aggressive interventions and escalating health costs, delaying unavoidable death may not influence patient outcome and often leads to reduced quality of life [5]. Cancer patients who die in a hospital typically experience more pain, stress, and depression than similar patients who die in hospice or at home [6,7]. Put differently, significant numbers of terminally ill patients may be suitable for and better served by palliative care but are nevertheless readmitted to acute hospitals multiple times [8].

Contributors to this disparity are multifactorial. On one hand, given the complex causal pathways to mortality, it can be difficult for doctors to decide the time and duration of the ultimate episode of decompensation, increasing the uncertainty to making appropriate treatment plans. On the other hand, quality-of-life discussion is associated with less aggressive medical care near death and earlier palliative care, which needs to be balanced with the will of patients to die with comfort, the expectations of families about satisfactory end-of-life care, and saving health resources if possible [5]. Also, mental illness or neurocognitive limitations are common in patients near the end of life, which further complicates assessment and decision making around care in this population [9].

To address this issue, prognostic tools have been developed to identify patients who are approaching a terminal state [10-16]. To date, however, there is no widely acceptable model for timely assessment and risk stratification of all-cause 1-year mortality that can be applied in the general population. Barriers to a widely applicable and accurate model include insufficient risk factors [16], incomplete data available in administrative datasets [17], and lack of generalizability of study patients. Knowledge gaps also exist with regard to the new challenges of social determinants of health (SDH) in terminally ill patients, in terms of the accessibility of health care resources, exposure to hazards, and knowledge of healthy behaviors [18]. Considering SDH in health care decision making could help care teams better target context-informed care, which fills a huge gap between hospital and hospice.

The objective of this analysis was to prospectively validate a machine-learning–based model to estimate a person’s risk of all-cause mortality in the next 12 months and assist care providers and families in decision making about appropriate care plans in the last few months of life [5]. The widespread use of electronic medical records (EMRs) affords a unique opportunity to understand health care status and improve care management at the population level. First, our study collected evidence from a rather comprehensive clinical profile, including demographics, medications, diagnoses and procedures, and radiology and laboratory test results for every patient. The breadth and richness of data allowed signals predicting mortality to be detected from the networked clinical patterns. Second, the methodology implemented XGBoost machine-learning techniques to extract valuable information from EMR datasets that could assign a predictive risk score to each individual [19]. Third, a large number of patients from the whole state ensured a certain degree of generalizability. Thus, this study was able to identify patients with diverse demographics and was readily translated to populations of different geographic origins and multiple social disparities.

We hypothesized that the past 12-month clinical histories of patients can be used to predict risk of all-cause mortality within the next 1 year. This prognostic model aims to provide an objective assessment to aid clinicians in decision making and counseling patients and their families about alternative treatments that incorporate their personal preferences and values. Of equal importance, identifying at-risk elderly patients and providing earlier palliative care may improve their quality of life and thus reduce futile utilization.

## Methods

### Reporting Method and Ethics Statement

The study was reported according to the Transparent Reporting of a Multivariable Prediction Model for Individual Prognosis or Diagnosis guidelines for a derivation and validation predictive model. Protected personal health information was removed for the purpose of this research. Analyzing deidentified data, this study was exempted from ethics review by the Stanford University Institutional Review Board (October 16, 2014).

### Health Information Exchange Dataset of Maine

Patients for this study were extracted from the Health Information Exchange (HIE) dataset, which covered records of nearly 95% of the population of the state of Maine and was managed by HealthInfoNet, an independent nonprofit organization. The data sources were EMRs collected from 35 hospitals, 34 federally qualified health centers, and more than 400 ambulatory practices in the state of Maine covering about 1 million patients [20,21].

We developed and applied the models using EMR data that included personal demographics, social determinants from the US Census Bureau, laboratory and radiographic tests coded according to Logical Observation Identifier Names and Codes, medication prescriptions coded according to the National Drug Code, and primary and secondary diagnoses and procedures that were coded using the *International Classification of Diseases, Ninth Revision, Clinical Modification*.

### Study Sample and Selection Criteria

The study included patients aged 65 years and older who visited any care facility in the Maine HIE network any time from September 5, 2013, to September 6, 2016.

Patients who died before September 5, 2014, were excluded. Those who did not have any active encounters during the 3 years before September 5, 2014, or whose zip codes were not located in Massachusetts, Maine, or New Hampshire were excluded from the study.

### Outcome Definition

A mortality case was defined as a coded date of death in the EMR database in the period from September 5, 2014, to September 4, 2015, in the derivation cohort and from September 5, 2015, to September 4, 2016, in the validation cohort.

### Predictive Factors of Mortality

**Figure 1:**
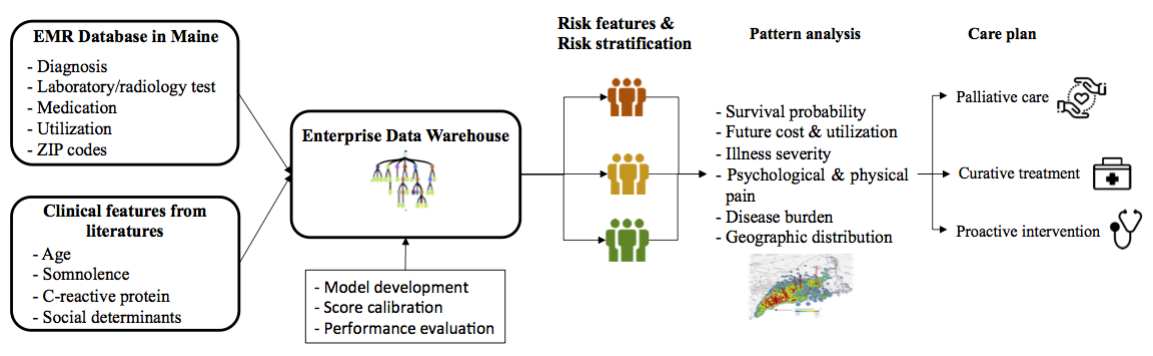
Study design.

A workflow chart is shown in Figure 1. The selection process was divided into 3 stages: univariate analysis, literature review, and XGBoost selection (Multimedia Appendix 1). Selection based on *P* values (*P*<.05, chi-square test or *t* test) was the initial screening process to trim down the high dimension of the dataset. Literature review was performed in parallel to identify risk factors of mortality that were identified by other studies. Those risk factors included demographics like age, chronic diseases (ie, cerebrovascular disease, cardiovascular disease, rheumatic disease), abnormal laboratory test results (ie, C-reactive protein, potassium), and medication prescriptions (ie, lactulose). Features identified by univariate analysis and literature review went into an XGBoost selection process, where the features were ranked based on their importance of predicting mortality in a model. To improve computational efficiency, we used machine-learning feature selection to determine the features that would go into the model prior to the derivation phase. Chronic disease history variables were modeled as dichotomous using primary and secondary diagnoses. Medication prescriptions were analyzed as the number of prescriptions for a particular medicine during the past 1 year. The thresholds defining laboratory tests as abnormal were set by facilities in the HIE network and treated as continuous variables.

We also assigned 8 SDH variables to each patient: percentage of the population residing in the zip code who were white, percentage of the population residing in the zip code who lived in a rural area, percentage of the population residing in the zip code who attained education at a bachelor’s degree level or higher, median household income in the zip code, unemployment rate in the zip code, Gini index of income inequality in the zip code, Social Vulnerability Index in the county (this is a measure of a community’s social conditions including socioeconomic status, household composition, minority status, and transportation), and Respiratory Hazard Index in the county (an indication of the adverse effects of pollutants).

These social determinants were mapped to the EMR database through a patient’s zip code and were categorized according to population quintiles (very low 0%-20%, low 20%-40%, medium 40%-60%, high 60%-80%, very high 80%-100%).

### Model Derivation

The derivation cohort was divided into 2 subsets for training and calibration. The initial model was derived based on the training subset: 99 features were input to describe the preceding 1-year clinical profile from September 5, 2013, to September 4, 2014, and the output was set to either 1 or 0 to indicate whether or not a patient was coded with mortality during the period from September 5, 2014, to September 4, 2015.

We adopted XGBoost and tuned the hyperparameters using grid search and cross validation. As a supervised machine-learning technique, it is able to discover statistical patterns in high-dimensional and multivariate data sets and handle nonlinear correlations and random errors both in input features and the output variable.

**Figure 2:**
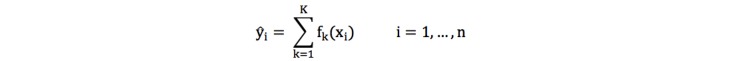
Final predictive estimate of the algorithm.

**Figure 3:**
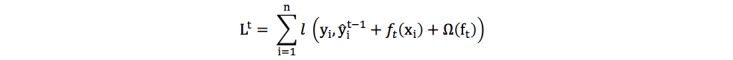
A sum of loss function and the overfitting control term.

During the process of model construction, the algorithm generated an ensemble of classification trees and ranked variable importance on the selection frequency of the variable as a decision node [22]. It then summed the scores in the corresponding leaves of each tree to calculate a final predictive estimate ŷ_i_ for the i-th (i = 1,...,n) instance, as demonstrated in Figure 2, where each f_k_ corresponded to an independent classification tree and K was the maximum number of trees in the algorithm. For our study, the depth of each tree was set to be 5 and K equaled 500. We protected against overfitting by penalizing the complexity of the algorithm. Parameters were adjusted to minimize the sum of loss function and the overfitting control term. The sum term at the *t* iteration was as seen in Figure 3, where l was a differentiable convex loss function that not only measured the difference between the target y_i_ and the prediction ŷ_i_^(t-1)^ of the *i* instance at the *t*-1 iteration but also took the f_t_ to improve the model most into account. The term Ω was set to penalize the complexity of the regression tree functions in avoid of overfitting. As a splitting method to grow trees, we used an approximate greedy algorithm, and features on each node were sorted to propose a couple of candidates at percentiles. Splitting points were chosen to optimize purity at the next level. The final predictive estimate was summed by individual trees.

In addition, a calibration subset was constructed to convert predictive estimates from the training set to positive predictive values (PPVs), a generalized risk measure with values that described the probability of mortality during the next 1 year. The PPV was calculated as the proportion of mortality in a subset of samples having predictive estimates higher than ŷ. In this way, all the predictive estimates were mapped to the calculated PPVs. Patients were then grouped into 3 categories: low risk, intermediate risk, and high risk based on calibrated scores. The relative risk of each patient was calculated as individual score divided by the mean score of all patients in the cohort (baseline). The relative risk indicated the probability of mortality during the next 1 year relative to the baseline.

### Model Validation

To test model performance, a validation cohort with clinical history from September 5, 2014, to September 4, 2015, was assembled to predict the risk of mortality from September 5, 2015, to September 4, 2016. The predicted score and relative risk for each patient were calculated. The predictive accuracy of the model was evaluated by calculating the area under the receiver operating characteristic (ROC) curves (discrimination) in both the derivation and validation cohorts, which reflected the ability to distinguish between patients at high and low risk of death. Clinical patterns and social determinants of patients in different risk groups were compared.

Prospective analysis of average and total cost in the year of death and the number of deaths by the top 22 mortality rate commodities in high-risk mortality patients were explored. This was because delaying unavoidable death often contributed to unsustainable and escalating health care costs due to aggressive and expensive interventions. In order to better allocate health care resources spent on treating high-risk patients at the end of life, we evaluated high-risk patients who died and analyzed associations between the cost of care in the last year of life and different chronic diseases. The 22 diagnoses were selected due to their associations with higher mortality among the high-risk patients in our database.

Additionally, studies have documented the escalating treatment cost and poor quality of life associated with significant burden of symptoms. In order to profile the seriously ill elderly population based on the debilitating diseases which may lead to death, we plotted the association between average cost and disease burden grouped by the top 20 chronic diseases of high-risk patients. All analyses were performed using R software (The R Foundation).

## Results

### Cohorts and Baseline Characteristics

**Figure 4:**
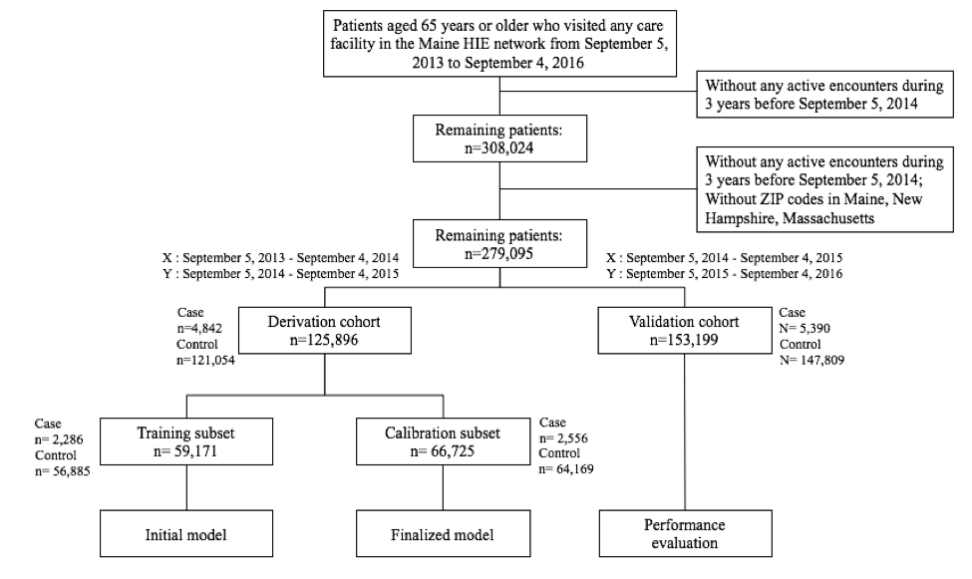
Construction of derivation and validation cohorts.

The final cohort included 125,896 patients for model derivation, 4842 of whom were recorded to have died in the next 1 year (from September 5, 2014, to September 4, 2015), and 153,199 patients for model validation, 5390 of whom died in the next 1 year (from September 5, 2015, to September 4, 2016). A cohort construction diagram is shown in Figure 4.

Table 1 shows the baseline characteristics for patients in derivation and validation cohorts. The 2 cohorts were evenly matched across demographics, payers, and clinical conditions (Table 1). Specifically, the study involved patients of balanced age (74.2 years in the derivation vs 74.3 years in the validation) and gender (57.64% [72,572/125,896] in the derivation and 57.56% [88,177/153,199] in the validation). With regard to clinical history, the occurrence of cancer and congestive heart failure, 2 well-established risk features of mortality, were present in 0.65% (989/153,199) and 1.09% (1667/153,199) of the validation cohort, respectively. Type 2 diabetes was present in 4.79% (7337/153,199) in the validation cohort.

### Significant Risk Features

Altogether, there were 14,680 features to profile each patient’s clinical history in the HIE dataset and socioeconomic status from the public data source. We identified 86 established clinical features of mortality from the literature review. In addition, 653 features survived after the univariate analysis and literature review. XGBoost used the approximate greedy algorithm to split trees by sorting and picking features on each node in order to optimize purity at each splitting level. Finally, a total of 99 features were selected as model predictors. The top 45 univariate features of mortality for elderly patients are shown in Table 2.

In accordance with previous studies, age (≥85 years) was recognized as the most impactful demographic feature in mortality risk. In our prospective analysis, the percentage of patients aged 65 to 74 years accounted for 59.25% (90,770/153,199) of the total population, and 25.60% (1380/5390) of all deaths. Comparatively, older adults (≥85 years) composed 12.62% (19,311/153,199) of the population and 37.35% (2013/5390) of all deaths. Furthermore, we analyzed the death rate of 4 priority noncommunicable diseases and 2 high-prevalence chronic diseases among different age groups (65-69 years, 70-74 years, 75-79 years, 80-84 years, and ≥85 years) (Multimedia Appendix 2). There was a significant rise in the percentage of death cases for cardiovascular disease and hypertension that presented in all patients aged ≥85 years. The percent of mortality cases of patients with chronic kidney disease diagnosis also increased nearly 10 times when comparing the 65- to 69-year age group with the 85-year age group. Cardiovascular disease showed the highest percentages of mortality cases in all age groups.

**Table 1:** Baseline characteristics.

Characteristic		Derivation cohort (n=125,896), n (%)	Validation cohort (n=153,199), n (%)	*P* value
**Age (years)**				.009
	65-74	73,989 (58.77)	90,770 (59.25)	—
	75-84	36,076 (28.66)	43,098 (28.13)	—
	≥85	15,831 (12.57)	19,331 (12.62)	—
Female		72,572 (57.64)	88,177 (57.56)	0.6
**Race**				<.001
	White	99,206 (78.80)	123,632 (80.70)	—
	Black	126 (0.10)	306 (0.20)	—
	Asian	19,010 (15.10)	20,682 (13.50)	—
	Other/unknown	7554 (6.00)	8579 (5.60)	—
Medicare		16,841 (13.38)	20,008 (13.06)	.01
Medicaid		263 (0.21)	341 (0.22)	.50
**Comorbid conditions**				.05
	Cancer	841 (0.67)	989 (0.65)	—
	Type 2 diabetes	6019 (4.78)	7337 (4.79)	—
	Renal disease	1911 (1.52)	2489 (1.62)	—
	Anemia	2879 (2.29)	3759 (2.45)	—
	Congestive heart failure	1386 (1.10)	1667 (1.09)	—
	Cerebrovascular accident/stroke	1747 (1.39)	2280 (1.49)	—
	Obesity	1465 (1.16)	1777 (1.16)	—

### Model Performance

Model outcomes of the derivation and validation phases are showed in Table 3. For derivation, the model had a c-statistic of 0.960 (see Multimedia Appendix 3 and Multimedia Appendix). Patients who died in the next 1 year (n=4842) had a mean relative risk of 30.91 (probability of mortality 30.91 times more than baseline). Among the 4842 patients, 595 were stratified as low risk, 1591 as intermediate risk, and 2656 as high risk. The mortality incidence and relative risk increased monotonically from low-risk (0.5%, 0.05) to high-risk (100%, 30.99) groups.

The model performance was slightly lower in the validation cohort (c-statistic 0.912), with 5390 patients who died in the next 1 year and a mean relative risk of 6.15. The total numbers of low-, intermediate-, and high-risk patients were 1384, 1593, and 2413, respectively, with high-risk patients accounting for 44.77% (2413/5390) of all patients who died within the next 1 year. The mortality incidence and relative risk climbed monotonically from low-risk (1.0%, 0.05) to high-risk (72.1%, 36.64) groups.

### Risk Stratification and Clinical Patterns

As demonstrated in Table 4, clinical patterns were compared among the low-, intermediate-, and high-risk groups in the validation cohort. There was an obvious difference in age distribution between the low-risk and other 2 groups. The average ages of low- and high-risk groups were 72 and 84 years, respectively. Survival analysis among the 3 risk groups showed the model to have good risk stratification in general (Multimedia Appendix 5).

In addition, patients in the high-risk group suffered from more severe comorbidities and used more health care resources. The proportion of patients with cancer of the bronchus (lung) in the high-risk group (6.54%) was much higher than in the low-risk group (0.11%) (*P*<.001). Chronic diseases such as type 2 diabetes, congestive heart failure, and chronic obstructive pulmonary disease (COPD) were present in 16.19% (542/3347), 19.18% (642/3347), and 16.61% (556/3347) of the high-risk group, compared with 3.04% (4273/140,344), 0.06% (82/140,344), and 1.46% (2055/140,344) of the low-risk group (*P*<.001), respectively With regard to laboratory tests, abnormal complete blood count, metabolic panel, urinalysis, and coagulation tests were present 48.07% (1609/3347), 51.33% (1718/3347), 24.59% (823/3347), and 11.95% (400/3347), respectively, of high-risk patients, while in the low-risk group, the percentages were much lower, at 0.18% (216/140,344), 0.27% (384/140,344), 0.06% (84/140,344), and 0.04% (58/140,344), respectively.

**Table 2:** Top 45 risk features in the final model with odds ratio and 95% confidence interval.

Category and differentiating features		Odds ratio	95% CI
**Demographics**			
	Age ≥85 years	1.41	1.06-1.48
**Social determinant**			
	Respiratory Hazard Index	1.24	0.92-1.40
	Unemployment rate	1.18	0.98-1.24
	Percent of population who lived in rural area	1.10	1.00-1.10
**Diagnosis**			
	Congestive heart failure	20.90	15.41-28.08
	Cancer of ovary	14.42	2.24-53.04
	Cancer of colon	14.07	10.08-19.08
	Cancer of stomach	13.64	3.26-86.57
	Cancer of bronchus, lung	12.38	2.91-36.04
	Chronic kidney disease	11.96	8.49-16.29
	Cancer of liver and intrahepatic bile duct	11.59	1.81-41.01
	Renal failure	11.22	8.88-14.06
	Cerebrovascular accident/stroke	9.31	5.59-14.68
	Cancer of brain and nervous system	8.65	2.07-24.4
	Rheumatic disease	6.15	3.85-9.12
	Myocardial infarction	6.13	5.21-7.29
	Leukemia	5.01	1.23-13.89
	Malnutrition	4.66	1.07-22.32
	Peripheral arterial disease	4.58	1.77-9.49
	Somnolence	2.99	1.85-4.43
	Cancer of breast	2.70	1.59-4.26
	Dementia	2.57	1.76-8.67
	Diabetes mellitus	1.43	0.36-2.22
**Laboratory test**			
	Hematocrit	4.13	2.00-6.31
	Potassium	3.55	2.50-4.76
	B-type natriuretic peptide	2.76	2.08-3.57
	Glucose	1.54	1.42-1.57
	C-reactive protein test	1.41	1.30-1.62
	Platelets	1.32	1.02-1.42
**Medication**			
	Pazopanib hydrochloride	3.66	1.92-10.65
	Lactulose	1.89	1.04-2.13
	Abiraterone acetate	1.85	1.34-2.45
	Metolazone	1.67	1.37-1.93
	Omeprazole	1.67	1.04-1.89
	Phenytoin sodium extended	1.61	0.96-1.78
	Furosemide	1.58	1.13-1.71
	Venlafaxine hydrochloride	1.54	0.98-1.63
	Clotrimazole	1.38	1.05-1.54
	Cephalexin	1.30	1.17-1.46
	Fluticasone/salmeterol	1.26	1.07-1.25
	Rifaximin	1.22	0.95-1.23
	Glipizide	1.19	1.07-1.36
	Olanzapine	1.13	1.00-1.69
	Carvedilol	1.10	1.07-1.13
**Utilization**			
	Inpatient days in the past 12 months	1.33	1.13-1.72

**Table 3:** Comparison of the model outcome in derivation and validation cohorts.

Outcome		Derivation cohort (n=125,896)	Validation cohort (n=153,199)
Died in the next 1 year, n (%)		4842 (3.84)	5390 (3.52)
**Risk score model**			
	Baseline score, mean (SD)	0.032 (0.035)	0.011 (0.072)
	Baseline score for mortality patients in the next 1 year, median (1st, 3rd quartile)	0.99 (0.11, 0.99)	0.067 (0.01, 0.34)
	Relative risk^a^ for mortality patient in the next 1 year, median (1st, 3rd quartile)	30.91 (3.48, 31.06)	6.15 (0.86, 31.42)
	Mortality risk category: low/intermediate/high	595/1591/2656	1384/1593/2413
**Percent incidence of mortality (95% CI)**			
	Low	0.50 (0.40, 0.60)	1.00 (0.80, 1.20)
	Intermediate	11.5 (11.0, 12.4)	16.80 (16.20, 17.52)
	High	100 (100, 100)	72.10 (71.50, 73.10)
**Relative risk for the population baseline (95% CI)**			
	Low	0.05 (0.04, 0.05)	0.052 (0.048, 0.055)
	Intermediate	2.76 (2.67, 2.88)	2.45 (2.41, 2.48)
	High	30.99 (30.9, 31.0)	36.64 (36.12, 37.07)

^a^Relative risk of each patient was defined as the ratio of the risk score of the patient to the baseline score (ie, the mean risk score of total population).

For high-risk elderly patients, we performed survival analysis for 4 leading causes of death defined by the World Health Organization: cardiovascular diseases, cancers, COPD, and type 2 diabetes. We found that all 4 chronic disease categories had a steep decrease in survival opportunity over time, indicating that our prognosis model aligned with current findings regarding major health burdens and high mortality among high-risk aged patients (Multimedia Appendix 6).

With respect to SDH, more high-risk patients lived in a community with high unemployment rate (29.46% [986/3347] vs 21.84% [30,646/140,344] in the low-risk cohort). Differently, 10.16% (340/3347) of high-risk patients had low median household income in their community, slightly lower than low-risk counterparts (17,560/140,344, 12.51%). Unemployment rate and education attainment (percentage of population who attained education at bachelor’s degree level or higher) contributed toward mortality risk in high-risk elderly patients, which made them more vulnerable to end-of-life care (see Multimedia Appendix 7).

For health care resource utilization, the mean cost during the last 12 months per patient in the high-risk group (US $10,575) was substantially higher than in the low-risk cohort (US $680). Seriously ill patients also used more health care resources as indicated by the greater number of outpatient visits at the end of life, 12 per patient per year, compared with 3 in low-risk patients.

**Table 4:** Clinical patterns of patients by risk categories in the validation cohort.

Characteristic			Low risk (n=140,344)		Intermediate risk (n=9508)		High risk (n=3347)
Age, years, median (1st, 3rd quartile)			72 (68, 78)		86 (80, 91)		84 (77, 90)
Female, n (%)			81,041 (57.74)		5356 (56.33)		1780 (53.18)
Race (white) , n (%)			113,678 (81.00)		7530 (79.20)		2510 (74.99)
**Diagnosis, n (%)**							
	Cancer of bronchus (lung)	163 (0.11)		60 (0.63)		219 (6.54)	
	Cancer of prostate	1306 (0.93)		97 (1.02)		92 (2.74)	
	Cancer of bladder	218 (0.15)		43 (0.45)		50 (1.49)	
	Cancer of breast	1052 (0.75)		65 (0.68)		63 (1.88)	
	Cancer of head and neck	138 (0.09)		18 (0.19)		14 (0.42)	
	Cancer of colon	68 (0.05)		23 (0.24)		49 (1.46)	
	Anemia	660 (0.47)		251 (2.64)		492 (14.70)	
	Pure hypercholesterolemia	5733 (4.08)		399 (4.19)		414 (12.37)	
	Type 2 diabetes	4273 (3.04)		468 (4.92)		542 (16.19)	
	Chronic kidney disease	266 (0.19)		114 (1.19)		285 (8.51)	
	Chronic liver disease and cirrhosis	434 (0.31)		52 (0.54)		104 (3.10)	
	Congestive heart failure	82 (0.06)		140 (1.47)		642 (19.18)	
	Chronic obstructive pulmonary disease	2055 (1.46)		483 (5.08)		556 (16.61)	
	Leukemia	57 (0.04)		4 (0.04)		24 (0.72)	
	Dementia	250 (0.17)		171 (1.79)		75 (2.24)	
**Community-level social determinant, n (%)**							
	Zip code with high median household income	17,560 (12.51)		1129 (11.87)		340 (10.16)	
	Zip code with high percentage of population who lived in rural area	86,577 (61.69)		5275 (55.48)		1962 (58.62)	
	Zip code with high unemployment rate	30,646 (21.84)		2140 (22.51)		986 (29.46)	
	Zip code with high percentage of population who attained education at bachelor level or higher	24,802 (17.67)		1634 (17.19)		720 (21.51)	
**Medication, n (%)**							
	Hypertension	27,962 (19.92)		5845 (61.47)		2681 (80.10)	
	Seizures	3775 (2.69)		952 (10.01)		584 (17.45)	
	Chronic obstructive pulmonary disease	4420 (3.15)		1410 (14.83)		902 (26.95)	
	Heart	11,897 (8.47)		3054 (32.12)		1622 (48.46)	
	Mental illness^a^	9144 (6.51)		2602 (27.36)		1352 (40.39)	
**Lab test, n (%)**							
	Abnormal complete blood count	216 (0.18)		523 (5.50)		1609 (48.07)	
	Abnormal metabolic panel	384 (0.27)		646 (6.79)		1718 (51.33)	
	Abnormal urinalysis	84 (0.06)		229 (2.41)		823 (24.59)	
	Coagulation test	58 (0.04)		77 (0.81)		400 (11.95)	
**Utilization, mean (1st, 3rd quartile)**							
	Cost past 12 months, US $	680 (340, 1360)		1700 (680, 4420)		10,575 (3230, 23,796)	
	Mean outpatient visit per 12 months	3 (1, 5)		5 (2, 10)		12 (6, 24)	

^a^Donepezil hydrochloride, lorazepam, prochlorperazine maleate, memantine hydrochloride, risperidone, haloperidol, paroxetine hydrochloride, rivastigmine, zolpidem tartrate, venlafaxine hydrochloride, temazepam, amitriptyline hydrochloride, olanzapine, and nortriptyline hydrochloride.

When we focused the analysis on high-risk patients with dementia, given the increasing attention to mental illness of terminally ill patients [23,24], we found a higher prevalence of dementia among high-risk elderly patients (2.24% [75/3347] vs 0.17% [250/140,344] in the low-risk cohort, *P*<.001). About 40.39% (1352/3347) of high-risk patients took medications for mental illness health conditions, substantially higher than in the low- (9144/140,344, 6.51%) and intermediate-risk (2602/9508, 27.36%) groups. We also compared mortality and health care use between high-risk patients with and without dementia (see Multimedia Appendix 8). Although the average ages in 2 groups were similar (82.8 vs 83.3 years), the mortality rate of patients who had dementia was slightly lower (41/75, 54.67%) than those at-risk patients without dementia (2372/3272, 72.49%) (*P*=.003). Dementia patients incurred less health care spending in the past 12 months (US $2795 vs $10,805) (*P*<.001) than patients without dementia and had a lower chronic disease burden (7.5 vs 10.9) (*P*<.001) and fewer inpatient days (1.3 vs 8.9), inpatient admissions (0.2 vs 1.3), and emergency department visits (1.1 vs 1.8) (*P*<.001). The community in which dementia patients lived was characterized by lower household income (US $40,407 vs $44,588) (*P*<.001).

### Average and Total Cost in the Final Year of Life of High-Risk Patients

**Figure 5:**
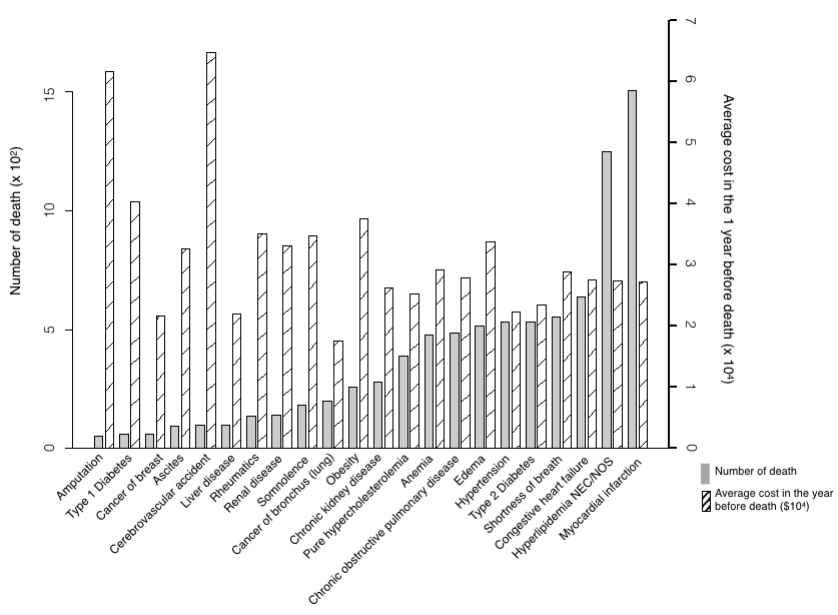
Prospective analysis of average cost in the year of death and the number of deaths by the top 22 mortality rate commodities in high-risk mortality patients.

**Figure 6:**
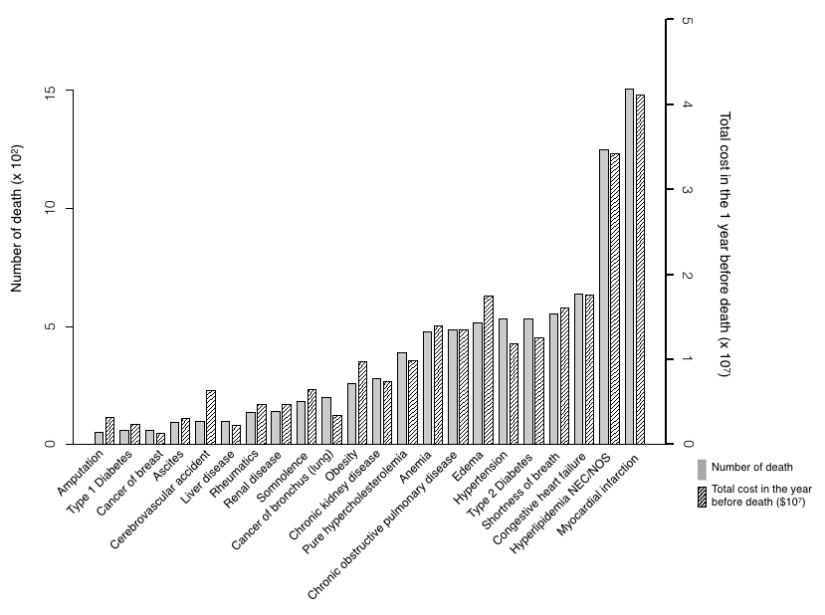
Prospective analysis of total cost in the year of death and the number of deaths by the top 22 mortality rate commodities in high-risk mortality patients.

Figures 5,6, and Multimedia Appendix 9 depict the average and total costs of medical care in the previous 1 year before death, which characterized the pertinent clinical profile and expenses of patients who died any time in the predictive year.

In the prospective cohort, the average cost in the last year of life overall was US $2346, and for the high-risk group it was US $21,799 (Figure 5). Among high-risk patients who expired, cerebrovascular accident, amputation, type 1 diabetes, obesity, and rheumatic diseases were the top 5 diseases in terms of 1-year average cost: US $64,756, $61,692, $40,329, $37,548, and $35,167, respectively. The percentages of high-risk patient deaths in those who had these conditions were 4.10% (99/2413), 2.15% (52/2413), 2.45% (59/2413), 10.73% (259/2413), and 5.64% (136/2413), respectively. This highlighted that elderly patients burdened with these diseases were likely to die with high attendant costs.

For 1-year total cost of mortality among high-risk patients (Figure 6), myocardial infarction, hyperlipidemia, congestive heart failure, edema, and shortness of breath were the top 5 diseases, given the absolute numbers of deaths in patients with these conditions in our study cohort. These diseases amounted to US $41,175,717, $34,227,212, $17,664,371, $17,495,202, and $16,063,029, respectively, and the percentages of high-risk patient deaths in those with these conditions were 62.45% (1507/2413), 51.72% (1248/2413), 26.52% (640/2413), 21.47% (518/2413), and 22.96% (554/2413), respectively.

### Associations Between End-of-Life Cost, Resource Utilization, and Disease Burden of High-Risk Patients

We found congestive heart failure (642/3347), COPD (556/3347), and type 2 diabetes (542/3347) were the 3 chronic diseases with the largest populations among these 20 diseases (see Multimedia Appendix 10). Patients with these 3 diagnoses had an average of 16.9, 14.0, and 16.1 chronic diseases. Patients with somnolence had the largest number of chronic diseases (20.1 per member) and the fourth highest average cost (US $34,378) of high-risk patients. Patients with leukemia were comorbid with nearly 18.5 chronic diseases at their end of life, with a high average cost of US $32,514 (compared to the overall average of US $21,799). The pattern differed from that of low-risk patients (see Multimedia Appendix 11).

Meanwhile, health care use also increased with end-of-life diseases (see Multimedia Appendix 12 and Multimedia Appendix 13). Among terminally ill patients, patients with somnolence and renal failure had the most inpatient admissions (2.6 times per member per year [PMPY]) and emergency department visits (3.4 times PMPY) at end of life. As mentioned previously, elderly patients with cerebrovascular accidents had relatively high mean cost in the final year of life as well as a high number of inpatient admissions (2.3 times PMPY) and a heavy disease burden (15.5 per member). For high-prevalence comorbid conditions, terminally ill patients with COPD, chronic kidney disease, and type 2 diabetes had a mean number of emergency department visits more than twice PMPY. Comparatively, high-risk elderly patients with dementia had fewer inpatient admissions (0.24 times PMPY) and emergency department visits (1.3 times PMPY) in their last year of life.

## Discussion

### Principal Findings

In an attempt to address the enormous treatment expenditures and unmet needs of patients approaching the end of life, we prospectively validated a tool to predict the all-cause 1-year mortality of statewide patients aged 65 years and older. To our knowledge, this study was the first to examine mortality of an elderly population with respect to end-of-life care, cost, and resource use.

Both the model outcomes and survival analysis supported the effectiveness and accuracy of our model in risk stratification of the high-risk group. The model performances in derivation and validation phases were excellent, with c-statistics of 0.960 and 0.912, respectively. It outperformed other models that were derived from limited numbers of risk factors or administrative data sources (see Multimedia Appendix 14). Further, the use of a statewide population in this study supported the generalizability of our findings to the nation as a whole.

The overall mortality in our study was 3.52% for elderly patients (65 years and older) in the state of Maine. The overall morality of the elderly population in 2015 was about 4.3% in the state of Maine [25], which was not far from the rate of our data pool.

Given that our cohort was highly imbalanced with 5390 cases and 147,809 controls derived from the 35 hospitals in the state of Maine with 1 million patients, the negative predictive values were expected to be high. However, our study goal was set to deliver robust PPVs (describing the probability of mortality within the next 1 year), and that can be challenging. Our model has prospectively identified the high-risk patients across the Maine state for population health, balancing the PPV and sensitivity requirements (see Multimedia Appendix 15, where PPV is 0.99% in the low-risk group versus 72.12% in the high-risk group).

Although some argued that conclusions in EMR studies may not be easily drawn [26], we have tried to improve the robustness of our study. First, our study is a prospective analysis originated from a dataset that covered records of nearly 95% of the statewide population in Maine. Unlike a randomized clinical trial (RCT) that has a well-designed sample cohort with targeted outcomes, our study was set to develop a predictive model using a statewide population with comprehensive clinical history including patient demographics, encounter history, vital signs, laboratory and radiology results, medication history, diagnoses, and procedures. Rather than providing a direct solution (ie, optimum treatment option) to each individual to extend life spans, our model intended to identify high-risk patients at early stages to provide early warning signals for improved health care quality and improved health care resource use.

Second, the active case-finding model and associated online real-time application were designed to track the evolving nature of total population risk of mortality in a longitudinal manner across all payers and diseases of elderly patients. Results were visualized on a real-time 24/7 online dashboard. This empowers the accountable care organization field staff and population health managers to visualize the risks derived from each resident’s historical medical records in the state of Maine. This tool is able to identify patient at high risk of 1-year mortality among the fragmented nature of population health information and improve quality of life and reduce futile treatment.

### Interpretation of Features

A list of risk factors survived after a feature selection process that integrated machine learning with clinical knowledge from the literature and practice (Table 2 and Multimedia Appendix 16). The importance of age to mortality risk was consistent with prior clinical studies and reflects a clinical scenario in which the association between older age and mortality may be explained by greater disease burden, associated complications, and functional impairment [27]. The age cutoff that best predicts mortality in elderly patients in our study and others is usually in the range of 80 to 85 years, an age group with a high prevalence of frailty, dependence, and geriatric syndromes according to related findings [28].

The model also highlighted the detrimental impacts of several chronic conditions, including congestive heart failure, kidney disease, cerebrovascular accident, rheumatic diseases, and myocardial infarction. Consistent with prior studies, we found congestive heart failure contributed significantly to the risk of 1-year mortality [11,29]. The illness trajectory of most heart and other organ system failure was distinct from that of cancers and comprised gradual decline, punctuated by episodes of acute deterioration and some recovery, with more sudden and seemingly unexpected death [30]. The hematocrit was the top laboratory test feature in this study and can indicate anemia or leukemia with lower values or lung and heart disease with higher values. Serum potassium levels can be associated with adverse outcome in patients with cardiovascular disease, with a U-shaped relationship between serum potassium levels and mortality in patients with acute myocardial infarction [31].

In our large cohort of elderly patients with various tumor sites, cancers of the ovary, colon, stomach, bronchus (lung), liver and intrahepatic bile duct, and brain and nervous system were the most impactful factors, independently associated with overall 1-year mortality. Overall, 70% of cancer deaths occur in patients older than 65 years, and for most cancers, there was a trajectory of steady progression and a clear terminal phase over a period of weeks, months, or years before death [30]. Factors such as age older than 80 years, functional impairment, mobility impairment, higher number of severe comorbidities, and malnutrition were common pathways that may increase the risk of cancer-related mortality [28]. Additionally, the adverse effect of metastatic status was greatest for breast and prostate cancers. Pazopanib hydrochloride, the most impactful medication in our model, is used to treat patients with advanced soft tissue sarcoma or gastrointestinal stromal tumors who have received prior chemotherapy.

In this study, dementia was found to increase mortality risk for elderly patients. As mentioned in prior studies, many features were reported as predictive of death in patients with dementia, including age, functional impairment, and disease severity [32]. However, it was likely that these patients had less accessibility to health care resources due to their cognitive impairment or that professional caregivers already viewed palliative care as appropriate for patients with end-stage dementia. Given the concentration of aging patients in the high-risk group, dementia as a test of the comprehensive geriatric assessment, especially for older surgical patients, can predict postoperative outcomes [33] and thus guide personalized approaches to medical care. Further, prescribing of antipsychotic medications for dementia patients was associated with higher mortality rates [34]. We also found that medications used to treat mental or mood disorders contributed to higher mortality probability, in accordance with prior literature. The side effects of psychotropic medications, particularly weight gain and impaired glucose tolerance, may increase the risk of excess mortality in people with mental illness [35]. It was also reported that conventional antipsychotics were associated with higher mortality than atypical antipsychotics [36]. These findings should give rise to more attention to mental illness in terminally ill patients, not only because of higher fatality rates from cancer in psychiatric patients [23] but also because of great opportunities to improve end-of-life care for these vulnerable patients, given their decreased ability to communicate need and the severe physical consequences [37].

Last, this study also featured SDH at the community level (zip code level), which was recently recognized as increasingly influential on morbidity and mortality [38-40]. Among several social determinant inputs, the Social Vulnerability Index and Respiratory Hazard Index were highly weighted. They reflected the degree to which a community exhibited certain social conditions (eg, high poverty or crowded households) and was exposed to pollution, respectively. Notably, patients with different racial or income backgrounds have been found to vary in their treatment preferences, advanced care planning, and access to health care resources [24]. Based on these findings, care providers may consider SDH information in their assessment of end-of-life medical care and prognosis.

### Planning for a “Good Death” in Terminal Phase

Death is inevitable, but there are a variety of ways to care for dying patients. A good death—“one that is free from avoidable death and suffering for patients, families, and caregivers in general accordance with the patients’ and families’ wishes” [41]—often optimizes the quality of life of terminally ill patients before a timely, dignified, and peaceful death.

Based on prospective validation of the statewide elderly population, the meaningful use of our model may be to stratify the population and identify patients at high risk of mortality, for whom timely targeted curative treatments may be indicated or palliative care plans may yield better quality of life and lower medical cost.

Planned treatments can be curative or palliative, depending on the diseases. Currently, the delivery of palliative care mainly targets malignant diseases. The trajectory of most cancers may be punctuated by the positive or negative effects of palliative oncological treatment. Most weight loss, impaired ability, and reduction in performance status for self-care occurs in patients’ last few months [30]. With earlier identification and open discussion about prognosis, there is time to anticipate palliative needs and plan for end-of-life care.

Early intervention (survival prognosis 6 to 24 months) by a palliative care team can help improve symptom control and satisfaction with psychosocial support and decision assistance [42]. Consequently, specialist palliative care is a recommended element of care for patients with cancer, especially cancers with poor survival rates. Positive benefits of specialist palliative care services in hospital teams, home care teams, and inpatient services have been documented [43]. Given the significant advances that have been made in the treatment of certain cancers, local health care systems need to ensure that these treatment advances are accessible in areas of high incidence. Care providers can also act on lifestyle choices to improve prevention, such as smoking, obesity, and diet, which have been identified as leading causes of cancer mortality [44].

Long-term limitations with intermittent serious episodes are typical among seriously ill patients with heart failure, chronic respiratory diseases, or other organ failure. Deteriorations are generally associated with admission to the hospital and intensive treatment. In this sense, advanced identification by the prognostic tool will contribute more to informing the timing of death and planning for terminal care in a preferred setting.

For many life-threating diseases like congestive heart failure, actions can be taken and conditions can be managed to help avoid escalating pain. The treatment aim of symptom relief held greater importance to physicians for elderly patients, while delay of death was thought to be more important for relatively younger patients, as suggested by an international survey [45]. For example, follow-up monitoring by specially trained staff, access to specialized heart failure clinics, and other multidisciplinary strategies appeared to be efficacious to improve outcomes for heart failure patients [46].

People who escape cancer and organ system failure may die at an older age of either brain failure (such as Alzheimer or other dementia) or generalized frailty of multiple body systems [47]. The disease course that dementia usually follows is one of prolonged and progressive disability, which makes identification of the terminal phase very difficult. Such patients may lose weight and show a variety of symptoms like depression and neurologic signs occurring in combination with declining reserve that can prove fatal. Despite the wishes of the majority of dementia patients and their families to die at home [48], many frail elderly patients with dementia are currently admitted to the hospital to die when terminally ill. The use of end-of-life care pathways in nursing homes is proving increasingly effective in preventing such admissions [7]. The prognostic tool helps identify patients with dementia who are approaching the end of life in order to plan care and make provisions for adequate terminal care. Educational and self‐study programs for care assistants in nursing homes appear to improve knowledge and attitudes regarding end-of-life care in dementia, and this knowledge appears to be maintained [49].

Some disease features of short-term death such as dementia were also significant determinants of quality of life [9]. As current research shows, there is a 30% higher mortality rate from cancer in patients with mental illness even though their incidence of cancer is no greater than in the general population [23]. In addition, some elderly patients with dementia have limited literacy and experience large disparities in health care access [50], while many primary care physicians lack competence in dementia care and access to valid assessment tools. Our predictive tool can assist care providers to address knowledge deficits and stratify at-risk patients with dementia for timely referral to specialist palliative care [51].

### Cutting Back on Medical Futility

More than 15.5 million Americans with a history of cancer were alive on January 1, 2016. The Agency for Healthcare Research and Quality estimated that the direct medical cost (total of all health care expenditures) for cancer in the United States in 2014 was US $87.8 billion [52]. The economic burden of cancer in the United States was substantial and expected to increase significantly in the future because of expected growth and aging of the population [53]. Consistent with the intensity of treatment for initial care, recurrence, and end-of-life care, costs of cancer were highest in the initial period following diagnosis and at the end-of-life stage [54]. In this study, seriously ill patients with cancer of the colon, blood (leukemia), stomach, and breast had relatively higher average cost in the final year of life, presenting as US $34,485, $32,514, $22,388, and $20,780, respectively, as well as high emergency department and inpatient resource use.

Medicalized deaths did not seem to be what cancer patients wanted, however. In a randomized controlled study, when patients with advanced cancer were given palliative care alongside standard treatment such as chemotherapy, the group receiving palliative care had lower rates of depression and were less likely to report pain [42]. In fact, increases in mortality incidence seen in the older patients (≥85 years) in the past may have been related to more aggressive diagnostic testing (eg, computed tomography imaging and stereotactic biopsy procedure) for this population [55], indicating potential overtreatment coupled with poor outcomes.

Although it was often assumed that dialysis will restore health, this was not always the case for some prevalent causes of death such as chronic renal disease, cardiovascular disease, and chronic respiratory disease. For example, despite improvements in survival among patients receiving maintenance dialysis over the past 2 decades, mortality rates in the end-stage renal disease population remained disturbingly high [56]. Older dialysis patients spend twice as many days in the hospital during the last month of life compared with Medicare beneficiaries with cancer. This indicates that when patients met dismal probability of survival and poor quality of life in the future, there was an opportunity to cut down the annual direct medical costs for end-stage renal disease, which are nearly US $28.6 billion [56].

The futility and discomfort of aggressive treatments combined with the underrecognition and undertreatment of pain in patients with severe dementia support the use of palliative care for advanced dementia [57]. Further, limited use of antibiotics has not been associated with increased mortality, and aggressive treatment of infections has not been shown to alter underlying disease processes [58].

Our prognostic model helps address the problem of futility by identifying patients who are receiving aggressive intervention but may benefit from being referred to palliative care at an earlier time. Should therapy fail and the patient choose not to continue with treatment, early referral to palliative care may be a benefit as well. Information from the prognostic model may stimulate an open conversation and provide evidence of why treatment is judged to be medically inappropriate, promoting the synchronization between medical teams, patients, and families [59].

### Limitations

This study has several limitations. First, compared to RCTs and other observational studies, the EMR-based study had real-life data challenges including missing or inaccurate values and sparse data. It is possible that some longitudinal clinical data were missing for certain patients in our EMR data warehouse, where the uncoded mortality cases could be outliers of the model and affect accuracy.

Second, the population aged 65 years and older in the United States has different distributions of race (white: 83.1% vs 80.7%; black: 9.1% vs 0.2%; Asian: 4.27% vs.13.5%) and comorbidities (cancer: 0.2% vs 0.6%; diabetes: 25.9% vs 4.7%) than the population of our study, which focused on older patients in Maine [60]. Recalibration and other necessary adjustments would be needed before leveraging the model validated with the population in the state of Maine to other regions of the United States. Using a cohort with race and comorbidity distribution similar to the United States, we shall build a more transferrable model.

Third, cost calculations were based on estimates from the literature rather than medical claims data. Using state cost averages that included items such as type of chemotherapy or laboratory costs, these estimates provided a justifiable approximation of the overall impact on 1-year cost.

### Conclusions

Our prognostic model, prospectively validated for identifying elderly patients at risk for mortality, had a good predictive ability and generalized well among the elderly population (≥65 years) in the state of Maine. We identified statistically significant and clinically meaningful risk factors to help predict mortality and support clinical decision making by grouping high-risk patients based on clinical history. This tool should be a valuable adjunct for helping patients make better quality-of-life choices and alerting caregivers to target better interventions and counseling to individuals at high risk for mortality.

## Appendix

Multimedia Appendix 1 Workflow of feature selection.

Multimedia Appendix 2 Percentage of deaths by diagnosis and age group in the state of Maine.

Multimedia Appendix 3 Receiver operating characteristic curve and c-statistics of derivation cohort.

Multimedia Appendix 4 Receiver operating characteristic curve and c-statistics of validation cohort.

Multimedia Appendix 5 Survival curves for elderly patients in low-, intermediate-, and high-risk groups.

Multimedia Appendix 6 Survival curves of mortality for high-risk patients with 4 main types of chronic diseases defined by the World Health Organization.

Multimedia Appendix 7 Odds ratio plot of social determinants for death in high-risk and low-risk groups.

Multimedia Appendix 8 Mortality and health care utilization among high-risk patients with and without dementia.

Multimedia Appendix 9 Total cost, average cost, and number of deaths among patients at high-risk for mortality (data in Figures 3 and 4).

Multimedia Appendix 10 Association between average cost and disease burden grouped by the top 20 chronic diseases among high-risk patients.

Multimedia Appendix 11 Association between average cost and disease burden grouped by the top 20 chronic diseases among low-risk patients.

Multimedia Appendix 12 Association between average number of inpatient admissions and disease burden grouped by the top 20 chronic diseases among high-risk patients.

Multimedia Appendix 13 Association between average number of emergency department visits and disease burden grouped by the top 20 chronic diseases among high-risk patients.

Multimedia Appendix 14 Details and performance of other clinical mortality prediction scores.

Multimedia Appendix 15 Risk stratification of derivation and validation cohorts.

Multimedia Appendix 16 Top risk feature importance (weights).

## Abbreviations

COPD: chronic obstructive pulmonary disease
EMR: electronic medical record
HIE: Health Information Exchange
PMPY: per member per year
PPV: positive predictive value
RCT: randomized controlled trial
ROC: receiver operating characteristic
SDH: social determinants of health
