# Supplementary figures and images for: Assessing Statewide All-Cause Future One-Year Mortality: Prospective Study With Implications for Quality of Life, Resource Utilization, and Medical Futility
Source: J Med Internet Res. 2018 Jun 4;20(6):e10311. doi: 10.2196/10311 (PMC6066632; doi:10.2196/10311)

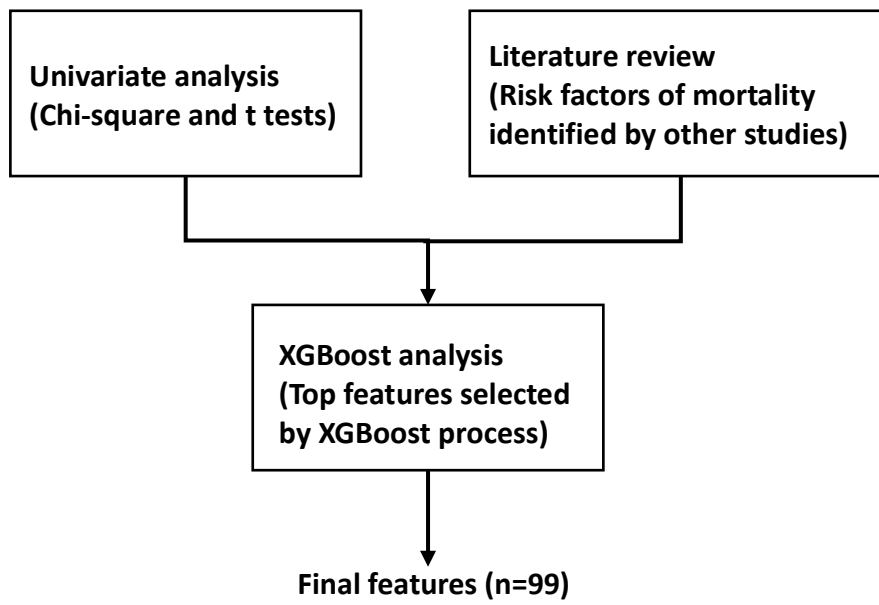

Multimedia Appendix. A workflow of feature selection.

Supplement: Multimedia Appendix 1 [file jmir_v20i6e10311_app1.pdf]

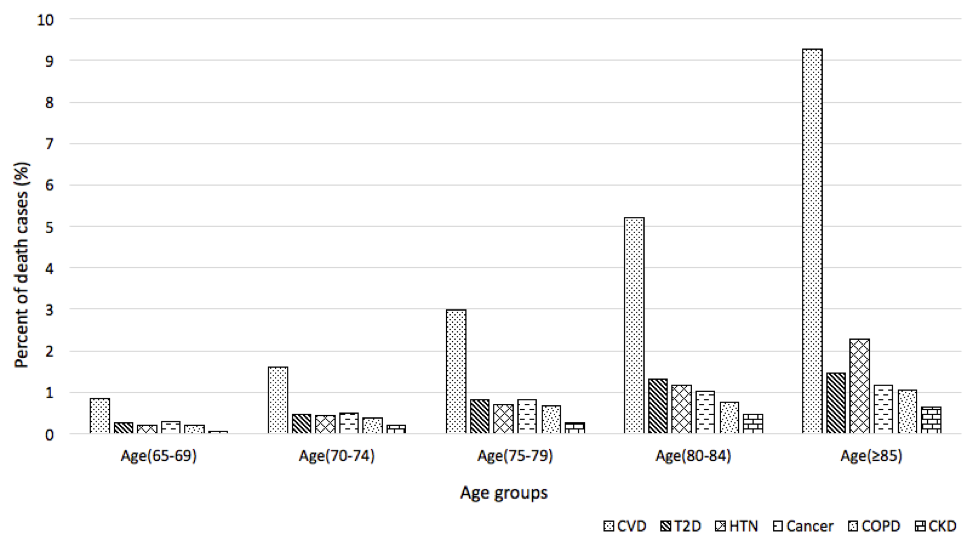

Supplement: Multimedia Appendix 2 [file jmir_v20i6e10311_app2.png]

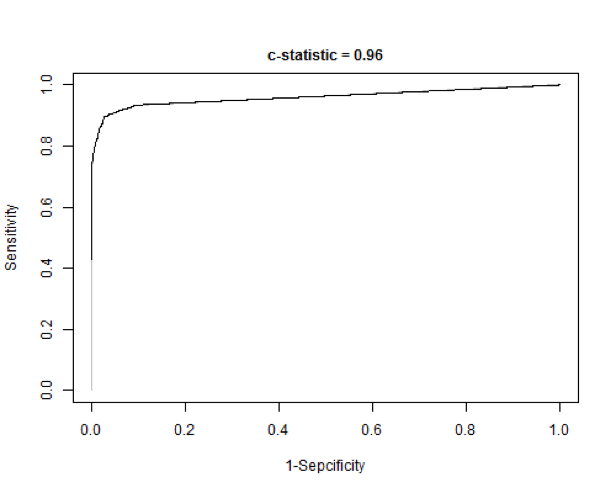

Supplement: Multimedia Appendix 3 [file jmir_v20i6e10311_app3.png]

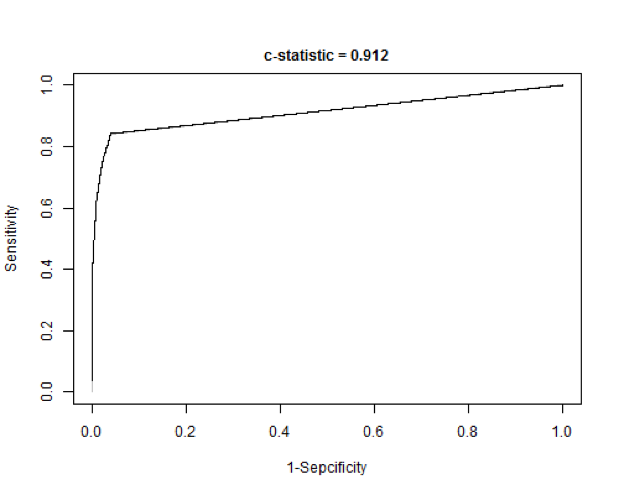

Supplement: Multimedia Appendix 4 [file jmir_v20i6e10311_app4.png]

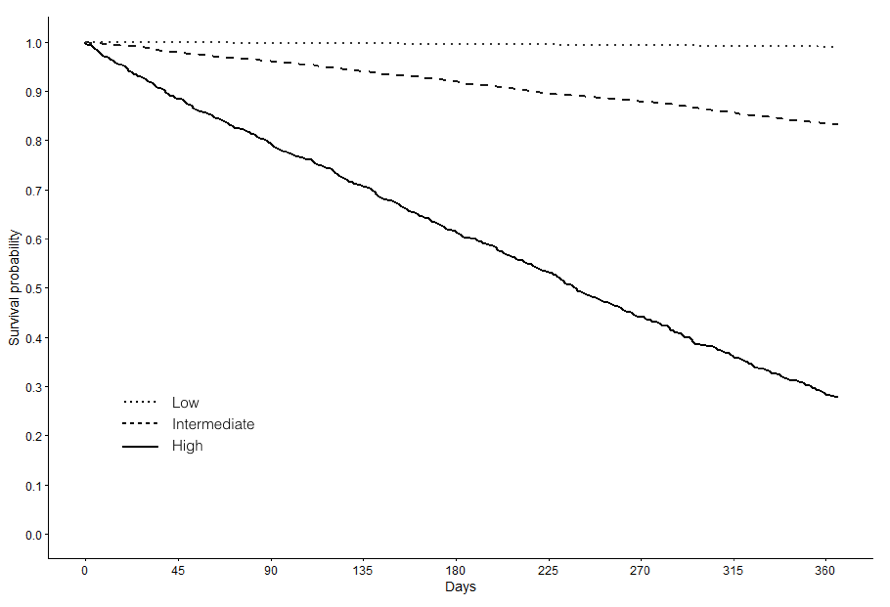

Supplement: Multimedia Appendix 5 [file jmir_v20i6e10311_app5.png]

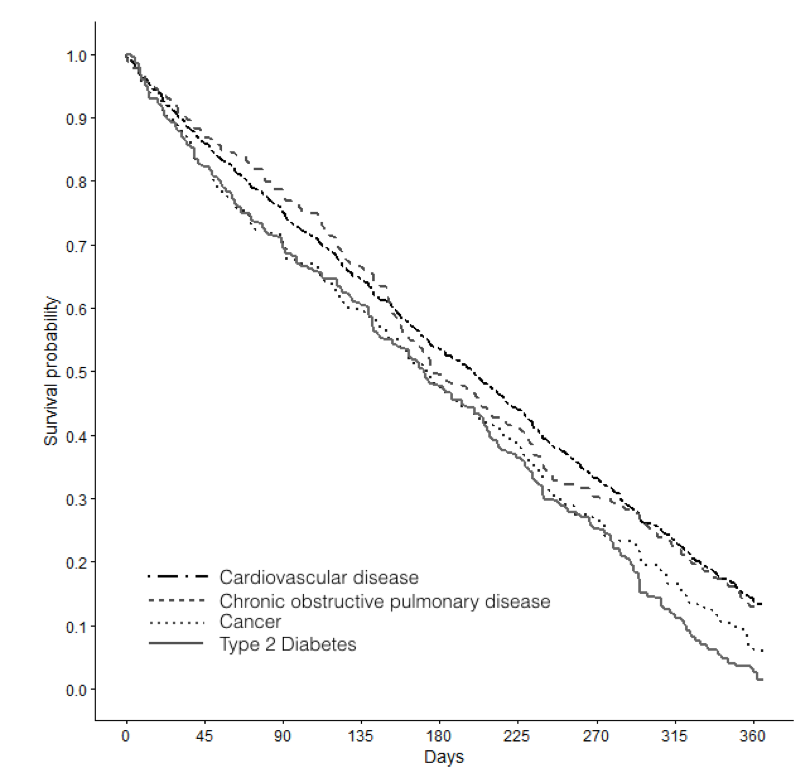

Supplement: Multimedia Appendix 6 [file jmir_v20i6e10311_app6.png]

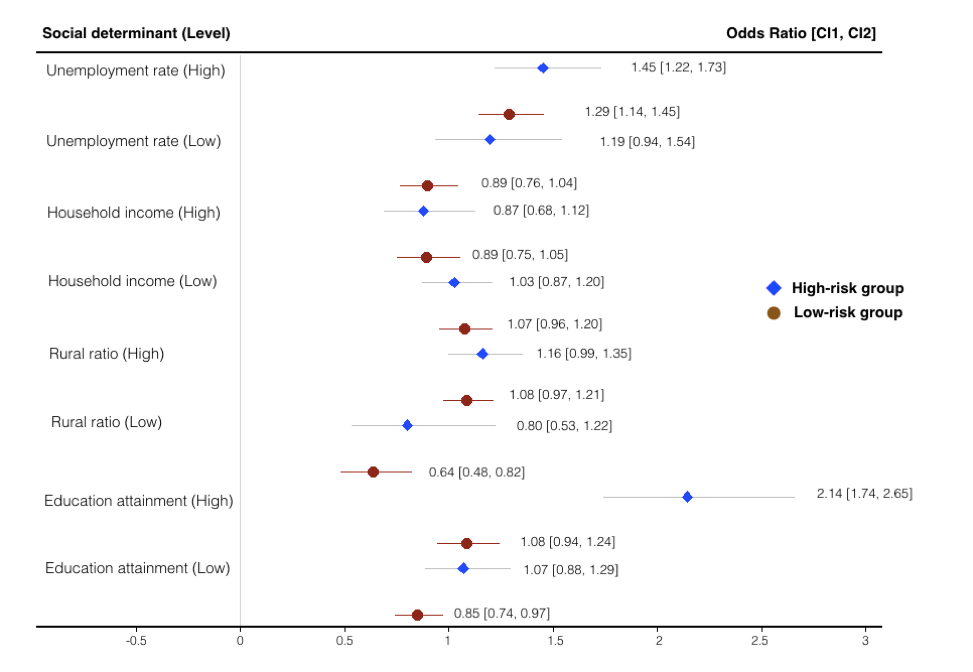

Supplement: Multimedia Appendix 7 [file jmir_v20i6e10311_app7.png]

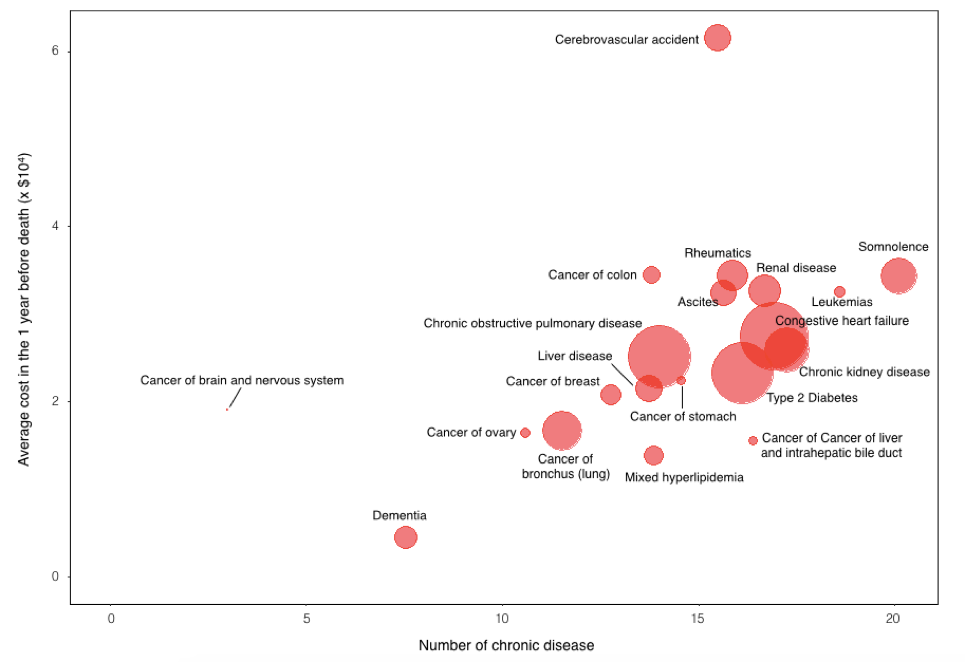

Supplement: Multimedia Appendix 10 [file jmir_v20i6e10311_app10.png]

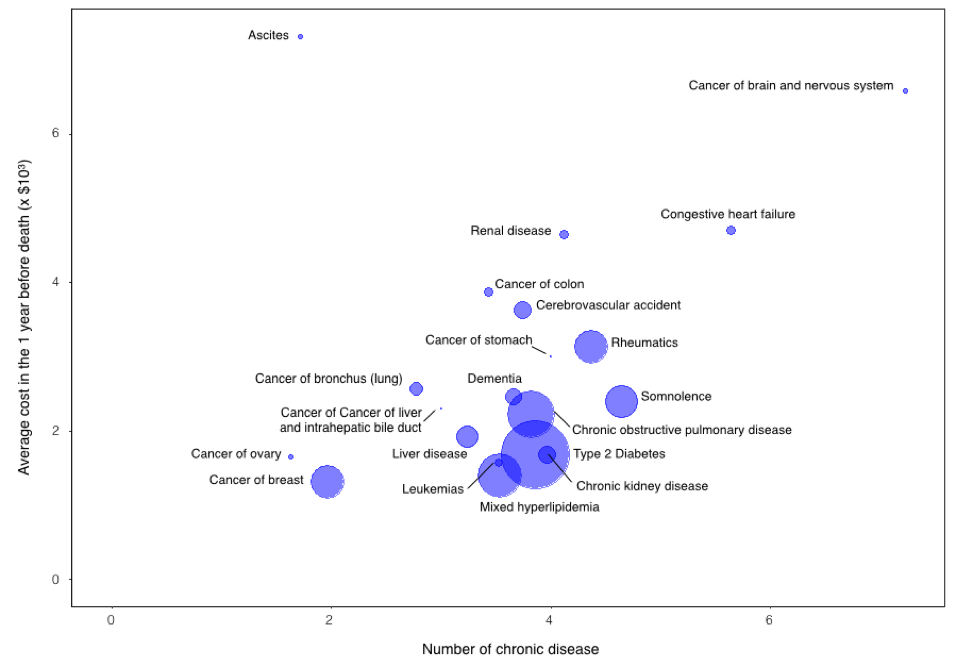

Supplement: Multimedia Appendix 11 [file jmir_v20i6e10311_app11.png]

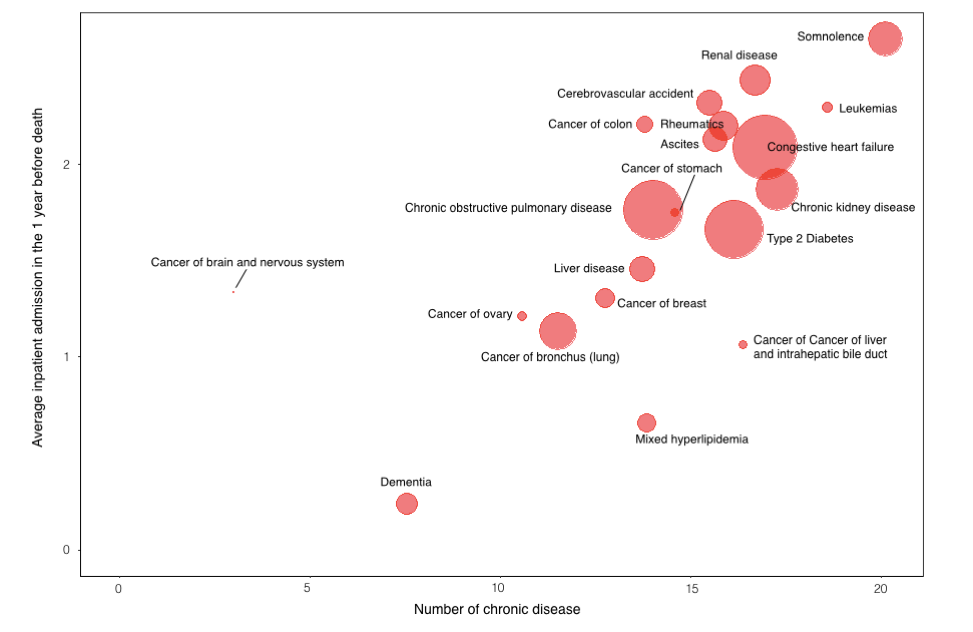

Supplement: Multimedia Appendix 12 [file jmir_v20i6e10311_app12.png]

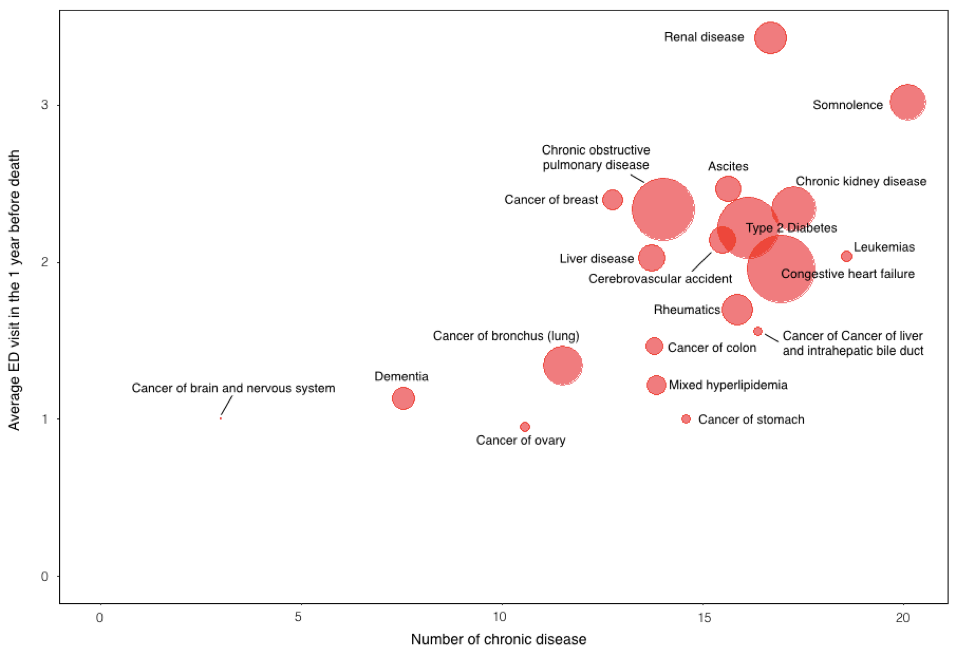

Supplement: Multimedia Appendix 13 [file jmir_v20i6e10311_app13.png]
