# Supplementary material for: Assessing Statewide All-Cause Future One-Year Mortality: Prospective Study With Implications for Quality of Life, Resource Utilization, and Medical Futility
Source: J Med Internet Res. 2018 Jun 4;20(6):e10311. doi: 10.2196/10311 (PMC6066632; doi:10.2196/10311)
Supplement: Multimedia Appendix 8 [file jmir_v20i6e10311_app8.pdf]

Multimedia Appendix 8

Mortality and healthcare utilization among high-risk patients with and without dementia

Outcome	Non-dementia N=3,272	Dementia N=75	p-value
Age	83.3	82.8	0.53
Number of deaths	2,372 (72.5%)	41 (54.6%)	0.003
**Utilization and disease burden**			
Cost in the past 12 months, $,	$10,805 ($3,380-$24,333)	$2,795 ($1,095-$5,080)	<0.001
Number of chronic diseases	10.9 (±7.4)	7.5 (±3.9)	<0.001
Inpatient days in the past 12 months, Days	8.9 (±14.2)	1.3 (±4.3)	<0.001
Inpatient admissions in the past 12 months	1.3 (±1.6)	0.2 (±0.5)	<0.001
Emergency department visits in the past 12 months	1.8 (±2.6)	1.1 (±1.5)	<0.001
**Social determinants of health**			
Median household income, $	$44,588 ($39,077-$53,737)	$40,307 ($36,911-$46,760)	<0.001
Percent of population who lived in a rural location, %	50.1 (25.7-100)	35.2 (25.7-69.5)	0.009
Unemployment rate, %	6.9 (5.6-8.7)	8.7 (6.6-9.2)	0.083
Percent of population who attained education at bachelor’s degree level or higher, %	48.2 (44.3-51.7)	48.4 (46.0-51.7)	0.11

Data are presented as mean (±standard deviation), median (1st quartile, 3rd quartile), or frequency (%)
