# Supplementary material for: Assessing Statewide All-Cause Future One-Year Mortality: Prospective Study With Implications for Quality of Life, Resource Utilization, and Medical Futility
Source: J Med Internet Res. 2018 Jun 4;20(6):e10311. doi: 10.2196/10311 (PMC6066632; doi:10.2196/10311)
Supplement: Multimedia Appendix 9 [file jmir_v20i6e10311_app9.pdf]

Multimedia Appendix 9

The total cost, average cost, and number of deaths among patients at high-risk for mortality (data in Figure 3 and 4)

Disease	Total cost	Average cost	Number of deaths
Amputation	3,208,033	61,692	52
Type 1 Diabetes	2,379,429	40,329	59
Cancer of breast	1,302,305	21,705	60
Cerebrovascular accident	6,410,847	64,756	99
Ascites	3,047,665	32,770	93
Liver disease	2,228,083	22,060	101
Rheumatic disease	4,782,780	35,167	136
Renal disease	4,737,690	33,130	143
Somnolence	6,447,789	34,852	185
Cancer of bronchus (lung)	3,508,794	17,632	199
Chronic kidney disease	7,394,549	26,315	281
Obesity	9,725,183	37,548	259
Pure hypercholesterolemia	9,885,665	25,347	390
Anemia	13,955,352	29,195	478
Chronic obstructive pulmonary disease	13,510,445	27,856	485
Hypertension	11,869,544	22,353	531
Edema	17,495,202	33,774	518
Shortness of breath	16,063,029	28,994	554
Type 2 Diabetes	12,574,152	23,503	525
Congestive heart failure	17,664,371	27,600	640
Hyperlipidemia	34,227,212	27,425	1,248
Myocardial infarction	41,175,717	27,322	1,507

Total cost: The total cost of all high-risk mortality patients with this diagnosis in the one-year before death.
Average cost: The average cost of each high-risk patient with this diagnosis in the one-year before death.
Number of deaths: The number of high-risk patients with this diagnosis who died.
