# Supplementary material for: Assessing Statewide All-Cause Future One-Year Mortality: Prospective Study With Implications for Quality of Life, Resource Utilization, and Medical Futility
Source: J Med Internet Res. 2018 Jun 4;20(6):e10311. doi: 10.2196/10311 (PMC6066632; doi:10.2196/10311)
Supplement: Multimedia Appendix 14 [file jmir_v20i6e10311_app14.pdf]

Multimedia Appendix 14

Details and performance of other clinical mortality prediction scores

Model	Cohort	Risk factors	C-statistic
Walraven et al, 2010[1]	Medical and surgical patients who admitted to the community (n=4812)	Age,	0.648
Donzé et al, 2016[2]	Patients aged 70+ (n=1495 in derivation and n=1427 in validation)	Male sex; number of dependent activities of daily living at discharge; congestive heart failure (CHF); cancer (solitary, metastatic); creatinine level; low albumin level.	0.72
Bate et al, 2014[3]	Patients with a primary diagnosis of acute stroke (n=7539 in derivation and n=5026 in validation)	Age, stroke, chronic pulmonary disease, coagulopathy, CHF, deficiency anemia, fluid or electrolyte disorders, lymphoma, metastatic cancer, and psychoses, hospitalization index, length of stay, and discharge location.	0.79
Our model	Patients from Maine HIE database (n= 125,896 for derivation and n= 153,199 for validation)	99 clinical variables including demographics, diagnosis, medication, laboratory test result, utilization and social determinant	0.912

1. van Walraven, Carl, et al. "Derivation and validation of an index to predict early death or unplanned readmission after discharge from hospital to the community." Canadian Medical Association Journal182.6 (2010): 551-557.
2. Donzé, Jacques D., et al. "International validity of the HOSPITAL score to predict 30-day potentially avoidable hospital readmissions." JAMA internal medicine 176.4 (2016): 496-502.
3. Bates, BE, Xie, D, Kwong, PL, Kurichi, JE, Ripley, DC, Stineman, MG: One-year all-cause mortality after stroke: A prediction model. PM&R, 6(6): 473-83, 2014.
