# Supplementary material for: Assessing Statewide All-Cause Future One-Year Mortality: Prospective Study With Implications for Quality of Life, Resource Utilization, and Medical Futility
Source: J Med Internet Res. 2018 Jun 4;20(6):e10311. doi: 10.2196/10311 (PMC6066632; doi:10.2196/10311)
Supplement: Multimedia Appendix 15 [file jmir_v20i6e10311_app15.pdf]

Multimedia Appendix 15

Risk stratification of derivation and validation cohorts

Derivation cohort

Risk bin	Low	Intermediate	High	Total
Number of patients	109,592	13,648	2,656	125,896
Number of deaths	595	1,591	2,656	4,842
PPV (%)	0.54	11.66	100	3.84
Sensitivity	12.28	32.85	54.85	100

Validation cohort

Risk bin	Low	Intermediate	High	Total
Number of patients	140,344	9,508	3,347	153,199
Number of deaths	1,384	1,593	2,413	5,390
PPV (%)	0.99	16.75	72.12	3.52
Sensitivity	25.67	29.55	44.76	100

Number of patients: The number of patients classified in this risk group.
Number of deaths: The number of patients with this diagnosis who died.
PPV: Positive predictive values. The probability that a person who is predicted the presence of a disease truly gets the disease.
Sensitivity: True positive rate. The proportion of positives that are correctly identified among all cases.
