# Supplementary material for: Assessing Statewide All-Cause Future One-Year Mortality: Prospective Study With Implications for Quality of Life, Resource Utilization, and Medical Futility
Source: J Med Internet Res. 2018 Jun 4;20(6):e10311. doi: 10.2196/10311 (PMC6066632; doi:10.2196/10311)
Supplement: Multimedia Appendix 16 [file jmir_v20i6e10311_app16.pdf]

Multimedia Appendix 16

Top risk feature importance (weights)

Category	Differentiating features	Weights
Demographics	Age	0.5850
Social determinant	Respiratory Hazard Index	0.0251
	Unemployment rate	0.0021
	Rural ratio	0.0005
Diagnosis	Rheumatic disease	0.0148
	Cancer of brain and nervous system	0.0109
	Congestive heart failure	0.0103
	Cancer of breast	0.0062
	Cancer of colon	0.0058
	Cancer of bronchus; lung	0.0058
	Cancer of stomach	0.0047
	Myocardial infarction	0.0032
	Cancer of ovary	0.0031
	Somnolence	0.0017
	Cerebrovascular accident/stroke	0.0016
	Malnutrition	0.0013
	Renal failure	0.0011
	Mixed hyperlipidemia	0.0009
	Chronic kidney disease	0.0009
	Cancer of liver and intrahepatic bile duct	0.0008
	Leukemia	0.0005
	Diabetes mellitus	0.0005
	Dementia	0.0004
Laboratory test	Potassium	0.0362
	Glucose	0.0019
	Hematocrit	0.0007
	B-type natriuretic peptide	0.0003
	Platelets	0.0002
	C-reactive protein test	0.0002
Medication	Furosemide	0.0581
	Metolazone	0.0334
	Haloperidol	0.0247
	Abiraterone acetate	0.0109
	Fluticasone/Salmeterol	0.0084
	Rifaximin	0.0084
	Lactulose	0.0069
	Clotrimazole	0.0069
	Omeprazole	0.0042
	Olanzapine	0.0042
	Cephalexin	0.0039
	Glipizide	0.0030
	Venlafaxine Hydrochloride	0.0025
	Phenytoin sodium extended	0.0021
	Pazopanib Hydrochloride	0.0016
	Carvedilol	0.0016

Utilization	Inpatient days in the past 12 months	0.0103
